# A Methodological Review of fNIRS in Driving Research: Relevance to the Future of Autonomous Vehicles

**DOI:** 10.3389/fnhum.2021.637589

**Published:** 2021-04-22

**Authors:** Stephanie Balters, Joseph M. Baker, Joseph W. Geeseman, Allan L. Reiss

**Affiliations:** ^1^Center for Interdisciplinary Brain Sciences Research, Department of Psychiatry and Behavioral Sciences, School of Medicine, Stanford University, Stanford, CA, United States; ^2^United States Navy, Washington, DC, United States; ^3^Department of Radiology, School of Medicine, Stanford University, Stanford, CA, United States; ^4^Department of Pediatrics, School of Medicine, Stanford University, Stanford, CA, United States

**Keywords:** fNIRS, functional near-infrared spectroscopy, autonomous driving, naturalistic brain imaging, methodology

## Abstract

As automobile manufacturers have begun to design, engineer, and test autonomous driving systems of the future, brain imaging with functional near-infrared spectroscopy (fNIRS) can provide unique insights about cognitive processes associated with evolving levels of autonomy implemented in the automobile. Modern fNIRS devices provide a portable, relatively affordable, and robust form of functional neuroimaging that allows researchers to investigate brain function in real-world environments. The trend toward “naturalistic neuroscience” is evident in the growing number of studies that leverage the methodological flexibility of fNIRS, and in doing so, significantly expand the scope of cognitive function that is accessible to observation via functional brain imaging (i.e., from the simulator to on-road scenarios). While more than a decade’s worth of study in this field of fNIRS driving research has led to many interesting findings, the number of studies applying fNIRS during autonomous modes of operation is limited. To support future research that directly addresses this lack in autonomous driving research with fNIRS, we argue that a cogent distillation of the methods used to date will help facilitate and streamline this research of tomorrow. To that end, here we provide a methodological review of the existing fNIRS driving research, with the overarching goal of highlighting the current diversity in methodological approaches. We argue that standardization of these approaches will facilitate greater overlap of methods by researchers from all disciplines, which will, in-turn, allow for meta-analysis of future results. We conclude by providing recommendations for advancing the use of such fNIRS technology in furthering understanding the adoption of safe autonomous vehicle technology.

## Introduction

**FIGURE 1:**
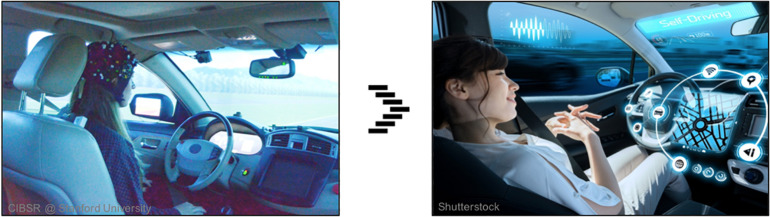
The future of automobile travel may remove control from the human driver all together, allowing drivers to become passengers who are able to engage in other, driving-unrelated tasks. Results from fNIRS brain imaging studies **(left)** will provide vital insights that may be integral in the design of future automated systems **(right)**. Written informed consent was obtained from the individual for the publication of any potentially identifiable images included in this article.

The era of autonomous driving is upon us. While semi-autonomous cars or “SAE Level 2 Automation Systems” (SAE, 2018) have become visible on our roads (Martinho et al., 2021; Tan et al., 2021), higher order automated systems (i.e., SAE Level 3–5 Automation Systems) are being engineered and tested (see Figure 1). Among the many advantages automated vehicles could bring to our streets is an increase in safety by outperforming the human through manipulation in driving dynamics (Goh et al., 2020), an increase in consumption efficiency (Phan et al., 2020), or a reduction of overall travel time by stabilizing traffic flow (Wu et al., 2018). However, until fully automated systems are engineered, approved, and legalized for the transport of (fully) passive passengers (i.e., SAE Level 4–5), the human driver will still manually drive the car, for at least segments of the drive, for many years to come. In fact, for Level 2–3 Automation, “fallback drivers” are needed to ensure that in the event the autonomous vehicle is unable to operate or experiences a failure, the driver can safely take-over and navigate the vehicle (SAE, 2018). The human operator must monitor the autonomous vehicle operations and its surroundings and, if possible, anticipate failures of the AV system, and respond quickly for potential take-over events.

Critically, it has been noted that drivers’ supervisions of automated vehicles are less than perfect fallbacks themselves. The simultaneous failure of the vehicle automation systems and the fallback driver can have disastrous consequences^1^. Research indicates that with the resumption of manual driving from lower levels of automation, drivers experience an increase in response time (Rudin-Brown and Parker, 2004) and in secondary task involvement (Winter et al., 2016). Further studies demonstrate that during periods of automation, drivers experience increased sleepy and drowsy behavior leading to decreases in driver vigilance (Miller et al., 2015). Thus, as long as a human is a necessary component of driving, a better understanding of the biological correlates to safe driver take-over events is critical to the development of SAE Level 2–3 autonomous vehicles.

**FIGURE 2:**
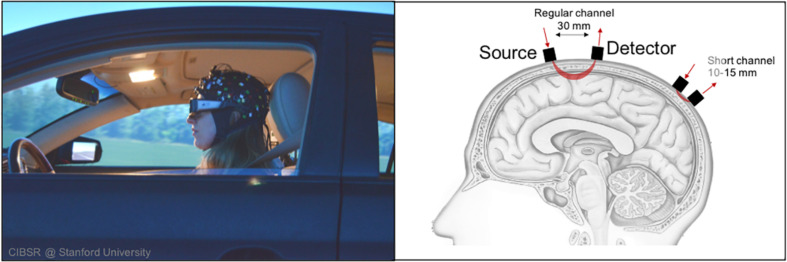
fNIRS’ methodological flexibility allows study of cortical activation during driving scenarios.

There has been increased effort in assessing driver state such as via physiological measurement tools (e.g., heart rate variability, skin conductance, etc.) or image recognition via board camera (e.g., drowsiness detection, emotion recognition, etc.) (Begum, 2013; Chowdhury et al., 2018). At the same time, there have been research efforts to elucidate the neuro-cognitive processes that underlie or precede these physiological or behavioral states via brain imaging (Kim et al., 2020; Ware et al., 2020). One brain imaging technique that has particularly gained traction in the past decade is functional near-infrared spectroscopy (fNIRS). In short, fNIRS is an optical brain imaging approach that uses near-infrared light to measure changes in oxygen levels within the cortex of the brain. As shown in Figure 2, a light source is situated next to a light detector approximately 3 cm apart. The emitted light travels in a banana shape path in all directions. As light passes through blood in the cortex, much of the light is absorbed by oxygen molecules attached to hemoglobin. The remaining light is detected and is used to calculate relative levels of oxygenated and deoxygenated hemoglobin within the region of the cortex between source and detector optodes. As previous research has shown, changes in cortical oxygenation (e.g., blood oxygen level dependence) occur when regions of the cortex become active (Strangman et al., 2002; Cui et al., 2011). Thus, by making such measurements quickly over time (e.g., ≥10 Hz), approximate real-time brain function may be observed and mapped to coinciding behavior.

Compared to electroencephalography (EEG), a commonly used functional neuroimaging approach that records electrical stimulation within the brain, fNIRS provides greater spatial resolution but slower sampling frequency (Scholkmann et al., 2014). Compared to functional magnetic resonance imaging (fMRI), the “gold standard” of functional brain imaging, fNIRS provides a faster sampling frequency but lower spatial resolution (Strangman et al., 2002; Cui et al., 2011). Thus, while maintaining a high sampling frequency, fNIRS also provides adequate spatial resolution needed to localize cortical brain function. Furthermore, fNIRS also affords many other methodological benefits, such as a tolerance to movement and methodological flexibility (Strangman et al., 2002; Cui et al., 2011). As it relates to the study of brain function and driving, fNIRS makes it possible to observe the neural correlates of driving in a manner that may not be feasible with other modalities (Tachtsidis and Scholkmann, 2016; Herold et al., 2017). In particular, recent advances in the portability of fNIRS systems have afforded neuroscientists the methodological flexibility to investigate neurocognitive behavior in naturalistic settings outside the MRI scanner (Baker et al., 2017; Yücel et al., 2021).

Though brain imaging research with fNIRS has reached a new era of *Real-Life Neuroscience* (Shamay-Tsoory and Mendelsohn, 2019; Holleman et al., 2020), it is important to note that the age of real-life neuroscience is still burgeoning, and that researchers are currently in the exploration phase of testing tools and methods to further strengthen our ability to study neurobiological signatures of more complex behavior in naturalistic environments such as driving. Furthermore, advances in technology and methodology have made fNIRS also accessible to groups outside of traditional neuroscience domains. For instance, engineers from diverse backgrounds such as Human Factors and Ergonomics, Human–Computer Interaction, and Engineering Design/Affective Engineering, have begun to study the neural signatures of drivers from their own research perspective (Solovey et al., 2009; Canning and Scheutz, 2013; Balters et al., 2017; Bosworth et al., 2019; Zhu et al., 2020). The transition between laboratory and the real-world (e.g., on-road) combined with advances from researchers outside of classical neuroscience (e.g., engineering) has resulted in a wide array of interesting and differing fNIRS methodologies. With respect to fNIRS driving research, “into the wild” and “multi-disciplinary” approaches have led to an eclectic mix of experimental designs, analytical techniques, and hardware configurations as highlighted throughout our review below. While such scientific diversity is expected in this early phase of any research domain, it will be essential for future research to minimize such methodological differences in a concerted effort to advance the field.

We argue that the construction of safe autonomous driving systems is an ongoing engineering challenge with high impact for society, and that an understanding of human behavior as part of this system is an important and open task. In complement with other psycho-physiological and behavior measures, fNIRS affords unique insights in understanding the underlying cognitive functions related to autonomous driving scenarios. To inform future autonomous driving studies, we therefore review the current state of fNIRS methodology pertinent to driving research. The overarching aim is to provide a methodological benchmark, which we can use to identify existing limitations that hamper fNIRS’s utilization in autonomous driving research. Specifically, we provide a detailed methodological review of the fNIRS-based studies of driving published prior to the year 2020. Throughout our review, we focus on seven relevant methodological domains that vary across studies: experimental environment; participant selection and documentation; task familiarization, physiological baseline; and inter-block/inter-trial intervals; task design and analytical approach; control task design; hardware specs, optode distance, and optode placement; and data processing and statistical analysis. For each of these domains, we identify potential best practices, summarize the approaches taken by researchers to date, highlight remaining hurdles and provide recommendations for future research. It is our hope that this paper will serve as an anchor for future discussion and collaboration within and between fNIRS researchers from different disciplines.

## Methodological Review

**FIGURE 3:**
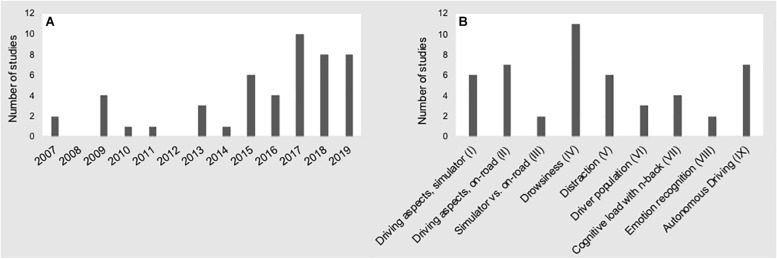
Number of fNIRS driving studies conducted from 2007 to 2019 **(A)**, and research topics **(B)**.

We executed a Google Scholar and PubMed search and considered all peer-reviewed manuscripts that were published through December 31, 2019. Our search strategy included the following keywords: “fNIRS car,” “fNIRS driver,” “fNIRS driving,” “NIRS car,” “NIRS driver,” “NIRS driving.” For each search, we inspected the first 250 entries for each keyword category and included all articles that met the criteria of “adult subjects,” “car driving,” and “simulator and/or on the road studies.” We operationally defined “simulator” as any virtual interface that included a physical steering wheel and pedals. As such, this allowed for a wide variation in simulator complexity^2^. Additionally, we checked the reference lists of the included articles for any additional relevant articles. We only included journal and conference publications in the English language. For instances in which the same content was published in more than one peer-reviewed publication (i.e., journal article and conference proceedings; *N* = 6), we distilled a key publication as representative in this review. From the initially identified 55 publications, a total of 48 publications met the above criteria. As shown in Figure 3A, the first studies in the field emerged about one decade ago, and about 5 years later fNIRS driving research significantly intensified. By means of thematic analysis of the identified publications we derived nine research topics of interest (see Figure 3B)^3^. In Table 1, we provide a brief summary of each paper including year of publication, author(s), research topics, basic manipulation and studied brain regions of interest. For an in-depth read on neural processes involved in driving, we refer the interested reader to following reviews (Liu et al., 2016; Lohani et al., 2019; Kim et al., 2020; Ware et al., 2020).

**TABLE 1:** Overview of driving research utilizing fNIRS.

References	Topic	Basic manipulation	Environment	*N* (of which *N* are women)	fNIRS instrument	Number of channels	ROI	Analysis approach
Harada et al., 2007*	II	7 km on-road driving around campus (young vs. elderly driver cohort).	On-road	28 (7)	NIRO-300, Hamamatsu Photonics	2	PFC	A
Shang et al., 2007*	I	Sim-driving without further stimuli.	Low-scale sim	1 (0)	Hitachi ETG-7100	66	PFC, OC	A
Li et al., 2009*	IV	3 h sim-driving vs. watching video of sim-driving (control group).	Low-scale sim	40 (NA)	TSAH-100, Tsinghua University	2	PFC	A
Shimizu et al., 2009*	I	Sim-driving with stop and go, narrow roads, and car-following.	Immersive sim	12 (NA)	OMM3000, Shimadzu Corporation	95	PFC, OC	A
Tomioka et al., 2009	VI	Sim-driving of Alzheimer’s Disease patients vs. healthy control group.	Low-scale sim	26 (0)	ETG-4000, Hitachi Medical Corporation	52	PFC	A
Tsunashima and Yanagisawa, 2009	IX	Sim-driving with and without adaptive cruise control.	Immersive sim	4 (0)	OMM3000, Shimadzu Corporation	42	PFC	A
Shimizu S. et al., 2011*	II	On-road driving on narrow roads in left and right hand vehicles.	On-road	16 (0)	Foire-3000 fNIRS, Shimadzu Corporation	32	PFC	A
Nakano et al., 2013*	VI	Sim-driving with “point and calling” when encountering traffic signs.	Immersive sim	3 (0)	Hitachi wearable optical topography	22	PFC	A
Yoshino et al., 2013a	II	On-road driving (day and night) with speed manipulations.	On-road	12 (4)	Foire-3000 fNIRS, Shimadzu Corporation	48	PFC, MC, PC	A
Yoshino et al., 2013b	II	On-road driving (day and night) with speed manipulations and u-turns.	On-road	12 (4)	Foire-3000 fNIRS, Shimadzu Corporation	48	PFC, MC, PC	A
Inoue et al., 2014*	III	Sim and on-road driving with turns at t-junctions.	Low-scale sim and on-road	8 (0)	Foire-3000 fNIRS, Shimadzu Corporation	24	PFC	A
Liu, 2014	IV	Sim-driving with and without maintaining speed control.	Low-scale sim	31 (4)	PocketNIRS, DynaSense Inc.	2	PFC	A
FakhrHosseini et al., 2015*	VIII	Detecting anger in participants during sim-driving.	Immersive sim	10 (4)	NIRO-200NX, Hamamatsu	4	PFC	FA
Khan and Hong, 2015	IV	35 min sim-driving to detect driver drowsiness.	Low-scale sim	13 (0)	DYNOT, NIRx Medical Technologies	28	PFC	LDA
Oka et al., 2015	I	Driving curves in the sim vs. watching video of curve driving in sim.	Immersive sim	15 (7)	Foire-3000 fNIRS, Shimadzu Corporation	48	PFC, pMC, MC, PC	A
Orino et al., 2015*	II	On-road driving on an express way with a sag.	On-road	11 (3)	Foire-3000 fNIRS, Shimadzu Corporation	48	PFC, pMC, MC, PC	A
Pradhan et al., 2015*	V	Sim-driving as single passenger and with passenger.	Low-scale sim	12 (0)	TechEn CW6	19	PFC	A
Unni et al., 2015*	VII	Five levels of n-back tasks while driving in the sim.	Low-scale sim	9 (NA)	NA	NA	PFC, PC	R
Ahn et al., 2016	IV	Well-rested vs. sleep deprived participants drive in the sim.	Low-scale sim	11 (1)	Custom-built system	8	PFC	A
Foy et al., 2016	VI	Sim-driving with overtaking tasks (novice/experienced and male/female).	Immersive sim	32 (NA)	fNIR 100, BIOPAC Systems Inc.	16	PFC	A
Nosrati et al., 2016	V	Sim-driving in non-distracted and distracted conditions.	Immersive sim	16 (11)	Custom-built system	2	PFC	A
Sibi et al., 2016*	IX	Autonomous sim-driving with three secondary tasks (i.e., reading).	Immersive sim	14 (3)	Device model 1100, Drexel University	16	PFC	A
Balters et al., 2017*	IX	Lane change in manual, partially, and fully autonomous sim-driving.	Immersive sim	28 (10)	NIRSport, NIRx Medical Technologies LLC	20	PFC	GLM
Horrey et al., 2017*	V	Sim-driving with boring and interesting auditory stimuli.	Low-scale sim	31 (13)	NIRO-300, Hamamatsu Photonics	2	PFC	A
Liu et al., 2017	II	On-road driving in simple driving and car-following conditions.	On-road	12 (0)	NirSan Danyang Huichuang Medical Equipment Co	16	PFC, MC, OC	C
Nguyen et al., 2017	IV	Sim-driving until experimenter detects signs of drowsiness.	Low-scale sim	11 (1)	Custom-built system	8	PFC	A
Orino et al., 2017*	II	On-road driving with steering and speed control.	On-road	6 (4)	Foire-3000 fNIRS, Shimadzu Corporation	98	PFC, MC, OC	A
Sibi et al., 2017*	IX	Sim-driving in manual, partially, and fully autonomous mode of operation.	Immersive sim	28 (10)	NIRSport, NIRx Medical Technologies LLC	20	PFC	GLM
Unni et al., 2017	VII	Five levels of n-back tasks while sim-driving.	Immersive sim	19 (2)	NIRScout, NIRx Medical Technologies LLC	78	PFC, OC, PC	R
Xu G. et al., 2017	V	Sim-driving with auditory distraction and visual vigilance tasks.	Low-scale sim	12 (5)	NirScan, Danyang Huichuang Medical Equipment Co.	36	PFC, MC, OC	C
Xu L. et al., 2017	IV	Baseline sim-driving versus solving arithmetic during sim-driving.	Low-scale sim	14 (NA)	NirScan, Danyang Huichuang Medical Equipment Co.	NA	PFC, MC, OC	C
Bruno et al., 2018	I	Driving curves in the sim, with correct and reversed steering.	Immersive sim	21 (10)	NIRSport, NIRx Medical Technologies LLC	40	PFC, PC	GLM
Chuang et al., 2018	IV	1 h sim-driving with reoccurring drifting events.	Immersive sim	16 (NA)	NIRScout, NIRx Medical Technologies LLC	18	PFC, MC, PC	FPA
Foy and Chapman, 2018*	I	Sim-driving on 4 different road types.	Immersive sim	30 (13)	fNIR 100, BIOPAC Systems Inc.	16	PFC	A
Huve et al., 2018*	I	Sim-driving in different weather and road types conditions.	Immersive sim	1 (NA)	WOT-220, Hitachi, Ltd.	22	PFC	ML
Ihme et al., 2018	VIII	Detecting driver frustration during sim-driving.	Immersive sim	16 (0)	NIRScout, NIRx Medical Technologies LLC	80	PFC	R
Le et al., 2018	VII	Sim-driving with auditory n-back tasks.	On-road	5 (1)	NIRS system Astem Corp.	4	PFC	R, ML
Li et al., 2018	IV	7 x 55 min sim-driving, followed by 5 min attention task.	Low-scale sim	13 (5)	Custom-built system	8	PFC	A
Yamamoto et al., 2018*	III	Encountering traffic signs in the sim and on-road driving.	Immersive sim and on-road	18 (10)	Foire-3000 fNIRS, Shimadzu Corporation	48	PFC, PC	A
Hidalgo-Munoz et al., 2019	IX	Sim-driving (manual and autonomous mode) while listening to radio.	Low-scale sim	12 (6)	NIRScout, NIRx Medical Technologies LLC	41	PFC, OC, PC	A
Huve et al., 2019 *	IX	3 min sim-driving in manual and autonomous mode of operation.	Immersive sim	1 (NA)	WOT–220, Hitachi, Ltd	22	PFC	ML
Khan and Hong, 2015	IV	30 min sim-driving to detect driver drowsiness.	Low-scale sim	5 (NA)	DYNOT, NIRx Medical Technologies	8	PFC	ML, LDA
Lin et al., 2019	IV	1 h sim-driving with reoccurring drifting events.	Low-scale sim	16 (4)	NIRScout, NIRx Medical Technologies LLC	26	OC, PC	A
Scheunemann et al., 2019	VII	Sim-driving in and outside of construction zones with n-back tasks.	Immersive sim	19 (2)	NIRScout, NIRx Medical Technologies LLC	78	Entire cortex	R
Sturman and Wiggins, 2019*	V	Sim-driving while being exposed to environmental cues/stimuli.	Immersive sim	62 (42)	NA	1	PFC	A
Tanveer et al., 2019*	IV	35 min sim-driving to detect driver drowsiness.	Low-scale sim	13 (0)	DYNOT, NIRx Medical Technologies	28	PFC	ML
Yamamoto et al., 2019*	V	Sim-driving and encountering traffic signs.	Immersive sim	12 (5)	Foire-3000 fNIRS, Shimadzu Corporation	48	PFC, PC	A
Zhu et al., 2019*	IX	Detecting brake intention during sim-driving.	Immersive sim	52 (10)	NIRScout, NIRx Medical Technologies LLC	41	PFC, PC, OC	ML

See Figure 3 regarding the definition of research topics. Below, we differentiate between “sim-driving” (i.e., simulated driving) and “on-road driving” (i.e., studies that were conducted on the road). The asterix symbol (*) marks research from the field of engineering. We utilize following abbreviations: region of interest (ROI), prefrontal cortex (PFC), motor cortex (MC), occipital cortex (OC), parietal cortex (PC), pre-motor cortex (pMC), block average (A), connectivity (C), factor analysis (FA), frequency power analysis (FPA), general linear model (GLM), linear discriminant analysis (LDA), linear or logistic regression (R), machine learning (ML), not applicable (NA).

### Experimental Environment

As highlighted in Table 1, researchers have used a myriad of approaches to study brain function under multiple driving conditions. This includes studies employing low-scale driving simulators (i.e., driving simulation conducted on desktop computer with small visual field; *N* = 17 studies) as well as a range of more immersive simulator environments (i.e., driver seated in a mock automobile with large visual field; *N* = 22 studies) and on-road conditions (*N* = 9). Simulated environments provide researchers with the ability to regulate and control the drivers’ experience. For instance, researchers may provide repeated instances of a specific driving task (e.g., diversion from unexpected obstacle) that may not occur frequently in real-world driving. Moreover, within simulated environments researchers may observe drivers in states (e.g., drowsiness) that would be too dangerous or irresponsible to observe in real-world driving. While simulated environments may require similar driver movements, many other mediating factors that affect the fNIRS signal during real driving are not present (Yamamoto et al., 2018). For example, imperfections in road conditions combined with road camber, centrifugal forces, wind, and unique characteristics of the automobile also introduce sources of noise that may not be easily captured in a driving simulator (Yamamoto et al., 2018). Another consideration regarding the task environment is the effect that the driving environment may have on the data. For example, the physical movements needed to operate low fidelity desktop-based simulated environments may differ greatly from immersive in-car experiences. In-car driving requires significant head and limb motions that have been shown to induce artifacts due to motion-induced optode shearing on the scalp (Huppert et al., 2009; Virtanen et al., 2011; Brigadoi et al., 2014). To help overcome such shearing, researchers may consider tightening the fit of the optodes on the participant’s head. It is important to note, however, that these measures will not completely remove artifacts due to motion, may increase participant discomfort over time, and may also introduce data artifacts of their own (Baker et al., 2017). Finally, ambient light in real-world driving is often much greater than in simulated environments and thus can negatively affect fNIRS signal compared to indoor lighting (Chenier and Sawan, 2007; Coyle et al., 2007; Baker et al., 2017). While these factors increase the difficulty of conducting fNIRS studies during on-road driving, they are often essential factors in an experimenter’s methodological design.

The majority of studies included here were conducted in a simulator setting, with only nine occurring in an on-road environment (Harada et al., 2007; Shimizu T. et al., 2011; Yoshino et al., 2013a, b; Inoue et al., 2014; Orino et al., 2015, 2017; Liu et al., 2017; Le et al., 2018). However, as described above, the quality of the simulators (e.g., fidelity of the visual environment, amount of visual field encompassed, realism of the simulator to a real automobile) varied between low fidelity desktop computer setups (Shang et al., 2007; Li et al., 2009, 2018; Tomioka et al., 2009; Liu, 2014; Khan and Hong, 2015; Pradhan et al., 2015; Unni et al., 2015; Ahn et al., 2016; Horrey et al., 2017; Nguyen et al., 2017; Xu G. et al., 2017; Xu L. et al., 2017; Hidalgo-Munoz et al., 2019; Khan et al., 2019; Lin et al., 2019; Tanveer et al., 2019) and more immersive simulated environments (Nakano et al., 2013; Oka et al., 2015; FakhrHosseini et al., 2015; Foy et al., 2016; Foy and Chapman, 2018; Huve et al., 2018, 2019; Sturman and Wiggins, 2019; Yamamoto et al., 2019) including large-scale simulators that comprise a real car mock-up along with a wide field of vision (Tsunashima and Yanagisawa, 2009; Shimizu et al., 2009; Orino et al., 2015; Nosrati et al., 2016; Sibi et al., 2016, 2017; Balters et al., 2017; Unni et al., 2017; Bruno et al., 2018; Chuang et al., 2018; Ihme et al., 2018; Zhu et al., 2019). To minimize motion artifacts, participants in eight studies were specifically instructed to avoid head (and limb) movements (Li et al., 2009; Takahashi et al., 2010; Liu, 2014; FakhrHosseini et al., 2015; Khan and Hong, 2015; Xu G. et al., 2017; Huve et al., 2018; Hidalgo-Munoz et al., 2019), potentially leading to a reduction in naturalistic behavior. Only two studies administered black coverage over the optodes to counteract artifact due to (sun-) light during on-road driving (Orino et al., 2015) and in the simulator (Sibi et al., 2016).

### Participant Selection and Documentation

Beyond assuring a sample size that is large enough for statistical interpretations, many participant characteristics (e.g., age, driving experience, gender, personality traits, etc.) have been shown to have a significant impact on driving behavior (Tao et al., 2017; Fountas et al., 2019). Participant selection and the reporting of participant characteristics, including but not limited to sample size, age, driving experience, and gender are important, as those factors may influence experimental design, analysis, and interpretation of the results.

Participant selection and information provided within the reviewed papers varied considerably. Across our reviewed papers, the number of participants ranged from one (Shang et al., 2007; Huve et al., 2018; Huve et al., 2019) to 62 (Sturman and Wiggins, 2019) participants with *M* = 16.81, *SD* = 12.37 across all studies. Only 75% (36 studies) reported the range, mean, and standard deviation of participant ages. Six studies reported solely a vague age range (e.g., “in their 20s”) (Shimizu et al., 2009; Tsunashima and Yanagisawa, 2009; Takahashi et al., 2010; Pradhan et al., 2015; Nguyen et al., 2017; Chuang et al., 2018), and the remaining six studies did not report participants’ ages at all (Nakano et al., 2013; Oka et al., 2015; Unni et al., 2015; Huve et al., 2018, 2019; Yamamoto et al., 2019). Only 18 studies reported driver statistics such as total number of years of driving and frequency of driving (Harada et al., 2007; Tomioka et al., 2009; Yoshino et al., 2013a, b; Oka et al., 2015; Orino et al., 2015, 2017; Pradhan et al., 2015; Foy et al., 2016; Nosrati et al., 2016; Xu G. et al., 2017; Bruno et al., 2018; Foy and Chapman, 2018; Li et al., 2018; Le et al., 2018; Hidalgo-Munoz et al., 2019; Scheunemann et al., 2019; Sturman and Wiggins, 2019). With respect to gender, eight studies did not report participants’ gender distribution (Li et al., 2009; Unni et al., 2015; Foy et al., 2016; Xu L. et al., 2017; Chuang et al., 2018; Huve et al., 2018, 2019; Khan et al., 2019), 12 studies examined exclusively male participants (Shang et al., 2007; Shimizu et al., 2009; Shimizu T. et al., 2011; Tomioka et al., 2009; Tsunashima and Yanagisawa, 2009; Nakano et al., 2013; Inoue et al., 2014; Khan and Hong, 2015; Pradhan et al., 2015; Liu et al., 2017; Ihme et al., 2018; Tanveer et al., 2019), and for the remaining 28 studies, the ratio of female to male participants ranged from 10 to 69%, with an average of *M* = 34.9% (*SD* = 17.6%). Notably, only three studies balanced the gender distribution (±1 person difference per gender group) across participants (Oka et al., 2015; Bruno et al., 2018; Hidalgo-Munoz et al., 2019).

### Task Familiarization, Physiological Baseline, and Inter-Block/Inter-Trial Intervals

Another study component is the activity used to familiarize participants with the driving environment. Participants who have not been familiarized with the driving environment may experience confusion with the system controls or may otherwise attend to factors outside of the task of interest. This, in turn, may elicit unwanted cortical activation that would not be present in the absence of such confusion. It is further advisable to establish a physiological baseline at the beginning of each scan, prior to the start of the experiment. This may be accomplished by simply instructing the participant to sit quietly and without movement for 30 s to 1 min. This can serve to stabilize the fNIRS signal, so that it is not artificially inflated or deflated due to excessive movement. Finally, it is important that experimental designs incorporate appropriately timed inter-trial and/or inter-block intervals. These intervals act to separate hemodynamic responses elicited during a task event or block prior to the start of the next trial or block. Event-related designs require jittered inter-trial intervals (i.e., randomized or pseudorandomized durations) so that the onset of the successive trial/block is unknown to the participant (Plichta et al., 2006, 2007).

Among the papers reviewed here, 23 did not report information regarding the driving familiarization task used. Participants were allowed to experience the driving environment (simulated or on-road) within 18 studies (Li et al., 2009; Shimizu et al., 2009; Liu, 2014; Khan and Hong, 2015; Pradhan et al., 2015; Oka et al., 2015; Ahn et al., 2016; Foy et al., 2016; Sibi et al., 2016; Horrey et al., 2017; Chuang et al., 2018; Ihme et al., 2018; Yamamoto et al., 2018, 2019; Khan et al., 2019; Lin et al., 2019; Sturman and Wiggins, 2019; Tanveer et al., 2019), and seven studies allowed participants to familiarize themselves with the driving environment and task-related stimuli (Tsunashima and Yanagisawa, 2009; Yoshino et al., 2013a, b; Unni et al., 2015; Balters et al., 2017; Sibi et al., 2017; Scheunemann et al., 2019). Only four studies reported a physiological baseline task (i.e., task designed to allow participants’ cortical activity to settle into a resting level), including sitting quietly with or without eyes open for a period of 2 min (Zhu et al., 2019), 5 min (Li et al., 2018), 10 min (Li et al., 2009), or 20 min (Xu G. et al., 2017). Establishing a resting level of oxygenation is an important component of fNIRS methodology, as it provides a baseline of blood oxygenation from which hemodynamic response magnitudes during the task are determined. Should no baseline be established researchers run the risk of missing true hemodynamic responses due to Type II (i.e., false negative) error. That is, detecting a rise in cortical oxygenation due to task demands (e.g., driving challenges) may be hampered if cortical blood oxygenation levels were artificially high to begin with. Generally, the recommended duration to establish a baseline is at least 30 – 60 s in which the participant sits quietly. Seven studies employed a block-design (Tsunashima and Yanagisawa, 2009; Nakano et al., 2013; Liu, 2014; Xu G. et al., 2017; Ihme et al., 2018; Hidalgo-Munoz et al., 2019). The inter-block duration ranged from roughly 30 s (Tsunashima and Yanagisawa, 2009; Liu, 2014; Hidalgo-Munoz et al., 2019), to a maximum of 5 min (Nakano et al., 2013; Xu G. et al., 2017; Ihme et al., 2018). In one study, a full day of rest between two block-conditions (i.e., drowsy vs. rested) was given (Ahn et al., 2016). Twelve studies used event-related design with inter-trial intervals ranging between 10 s and 1 min (Shimizu et al., 2009; FakhrHosseini et al., 2015; Oka et al., 2015; Unni et al., 2015, 2017; Nosrati et al., 2016; Balters et al., 2017; Sibi et al., 2017; Bruno et al., 2018; Chuang et al., 2018; Lin et al., 2019; Scheunemann et al., 2019).

### Task Design and Analytical Approach

The analytical approach that a researcher intends to take with their study is inherently related to methodological elements such as trial number and number of experimental conditions (Plichta et al., 2006, 2007). For instance, an adequate number of trials are required for each condition to provide a normal distribution of fNIRS samples, which is in turn required to identify a true effect from background noise. If the number of repetitions is too low the researcher will be more prone to both Type I (false positive) and Type II (false negative) errors because outlying values have a greater impact on smaller distributions. This means that single-trial studies may not be suitable for trial-based study designs. Researchers may obtain effect size estimates (e.g., Cohen’s *d*) from published reports and use such information to estimate the appropriate number of trials needed to obtain a desired statistical effect. Moreover, researchers may rely on online tools (e.g., Optseq) designed to aid in the methodological development of neuroimaging studies with respect to statistical power. As with other neuroimaging methods, fNIRS studies are typically conducted as block or event-related designs.

Block design studies require participants to engage in a task for at least a duration long enough to observe an entire hemodynamic response to a given stimulus or experimental condition. For example, a researcher interested in the effect of talking on a cellphone while driving may require participants to drive a pre-defined course 10 times while talking on the phone, then again without talking for a total of 20 trial blocks. Assuming, for the sake of our example, that talking on the phone did elicit cortical activity that was captured by fNIRS, such activity may be observed as a rise in oxygenation that occurs shortly after the beginning of each talking block and lasting until talking ended. The approximate duration required to observe a hemodynamic response function (HRF) is at least 10 s (Strangman et al., 2002; Cui et al., 2011), meaning that each task block must be at least 10 s in duration, although additional time is required to also observe the decrease of the HRF as cortical activity returns to resting levels. While an HRF may not be easily identifiable in a single block of the task, averaging each talking block together may reveal such a response. Several metrics [e.g., area under the curve (AUC), max/min value, etc.] derived from the block-averaged time series may then be calculated and used as the primary dependent variable for group-level analyses. The AUC for talking blocks may be expected to be greater than for non-talking blocks, which may be tested using common inferential statistics (e.g., Student’s *t*-test). However, because a hemodynamic response may vary for any number of reasons (e.g., attentional shift), the use of block durations that are significantly longer than a single HRF (e.g., 60 s or longer) may include unrelated cortical activity when averaging. Thus, researchers should consider limiting their block durations or parsing excessively long blocks into discrete sections for block averaging.

One well established alternative to block design tasks is the generalized linear modeling approach (GLM), which attempts to model small portions of an expected hemodynamic response through convolution onto the recorded fNIRS data. This is done by time-locking the onset and duration of each task trial to the fNIRS timeseries. If the onset of a trial induces an expected hemodynamic response, the fit of the GLM will be greater compared to conditions that do not elicit a hemodynamic response. Because the entire HRF is not sought, researchers may present multiple trials that are shorter in duration compared to block averaging. Furthermore, trials from different conditions may be pseudo-randomized and jittered so that all conditions are experienced evenly throughout the study, yet their onsets may not be reliably determined. Ultimately, the GLM approach will calculate standardized beta weights for each condition, which quantify the degree to which each condition elicited an increase (positive beta) or decrease (negative beta) in cortical response. Task- and control-condition beta weights may then be contrasted and used as a primary dependent outcome. Finally, multiple advanced statistical approaches (e.g., machine learning, functional connectivity, etc.) have been developed for fNIRS data that may also provide greater methodological flexibility. For instance, unconstrained machine learning analyses seek to identify unique patterns of cortical responding that occur during naturalistic driving. This approach may be best suited for long durations of real-world driving that do not include explicit trials. Similarly, while also amenable to trial- based task structures, functional connectivity analyses may be used to identify inter- or intra-brain communication that occurs during naturalistic driving.

As may be expected, the analytic approach employed by the studies reviewed here differed greatly. For instance, 60% (*N* = 29) used an averaging approach in which the fNIRS time series was averaged across a trial and/or event block. The average duration ranged from short blocks (i.e., 10–32 s) (Shimizu et al., 2009; Shimizu S. et al., 2011; Oka et al., 2015; Pradhan et al., 2015; Nosrati et al., 2016) to longer blocks (i.e., 40 s – 8 min 30 s) (Shang et al., 2007; Tomioka et al., 2009; Tsunashima and Yanagisawa, 2009; Nakano et al., 2013; Ahn et al., 2016; Foy et al., 2016; Sibi et al., 2016; Horrey et al., 2017; Nguyen et al., 2017; Foy and Chapman, 2018; Li et al., 2018; Hidalgo-Munoz et al., 2019; Sturman and Wiggins, 2019). Two studies opted to bin a long time series (i.e., 15 s to 60 min) into multiple “chunks” (Li et al., 2009; Lin et al., 2019). The remainder of these studies provided no information about the duration (Harada et al., 2007; Yoshino et al., 2013a, b; Inoue et al., 2014; Liu, 2014; Orino et al., 2015, 2017; Yamamoto et al., 2018, 2019). Nineteen studies employed analytical approaches that may be considered more complex than block averaging including GLM (Balters et al., 2017; Sibi et al., 2017; Bruno et al., 2018), functional connectivity (Liu et al., 2017; Xu G. et al., 2017; Xu L. et al., 2017), linear regression (Unni et al., 2015, 2017), logistic regression (Ihme et al., 2018; Scheunemann et al., 2019), machine learning (Huve et al., 2018, 2019; Khan et al., 2019; Tanveer et al., 2019), frequency power analysis (Chuang et al., 2018), factor analysis (FakhrHosseini et al., 2015), and linear discriminant analysis (Khan and Hong, 2015).

### Control Task Design

The objective of a control task is to provide a condition that is nearly identical to the primary task yet lacks the component that is expected to elicit a cortical response of interest. For instance, for a hypothetical study of the effect of distraction on the neurobiological signatures of driving, participants may find themselves actively driving the same course in the “distraction” and “no distraction” conditions. However, within the “distraction” condition an attentionally demanding task is added to the driving experience. These conditions allow the researcher to contrast cortical activation during distraction with activation under identical conditions save for the distracting component. The optimal control task will elicit activation from the same brain regions (e.g., motor cortex), but not the primary experimental region of interest. Thus, as opposed to contrasting activation during a task that requires movement with rest, it may be more appropriate to employ a control task that also requires movement. In other words, if our paradigm is to study distraction during *manual driving*, then the non-distraction control condition should also include manual driving to account for cortical activation related to the driving task itself (e.g., motor cortex, spatial processing, etc.). If, on the other hand, the aim is to derive neurocognitive signatures of distraction during *autonomous driving*, then the control task ought to include full automation as well. When selecting a control task, it is helpful to first evaluate brain regions that are expected to be active during the task of interest, including regions such as motor cortex that may not be of relevance to the primary experimental hypotheses yet may show activity due to motion when responding. The same concept applies to the design of the distraction task itself. Since driving requires a variety of different cognitive functions (e.g., motor planning, spatial processing, temporal processing, etc.), it is important for the distraction task to utilize similar cognitive functions. From a “statistical power” perspective it is therefore desirable to include a distraction task that predominantly utilizes other cognitive functions (e.g., auditory stimuli). Other research questions, however, might be tied to ecologically valid scenarios that require similar cognitive functions to the baseline driving task (e.g., use of visual GPS during driving). In these cases, an increase in the number of trials might provide high enough effect size. It is up to the clever experimenter and extensive piloting to identify control tasks that remain ecologically valid while satisfying statistical power criterion. In general, because the cognitive and physiological state of a participant may be expected to change over the course of an experiment, the presence of control trials throughout the task are important.

Control tasks used in reviewed fNIRS driving studies varied greatly from active driving tasks to passive resting states to studies that did not include a control task in their study design. While the majority (*N* = 37) employed an active control task (e.g., “baseline” driving without an experimental stimuli); six studies employed a passive control task ranging from resting states (Shang et al., 2007; Tsunashima and Yanagisawa, 2009; Oka et al., 2015; Huve et al., 2019; Zhu et al., 2019) to monitoring autonomous driving in the simulator (Bruno et al., 2018). Five studies did not include a control task in their study design (Shimizu et al., 2009; Inoue et al., 2014; Khan and Hong, 2015; Nguyen et al., 2017; Tanveer et al., 2019).

### Hardware Specs, Optode Distance, and Optode Placement

**FIGURE 4:**
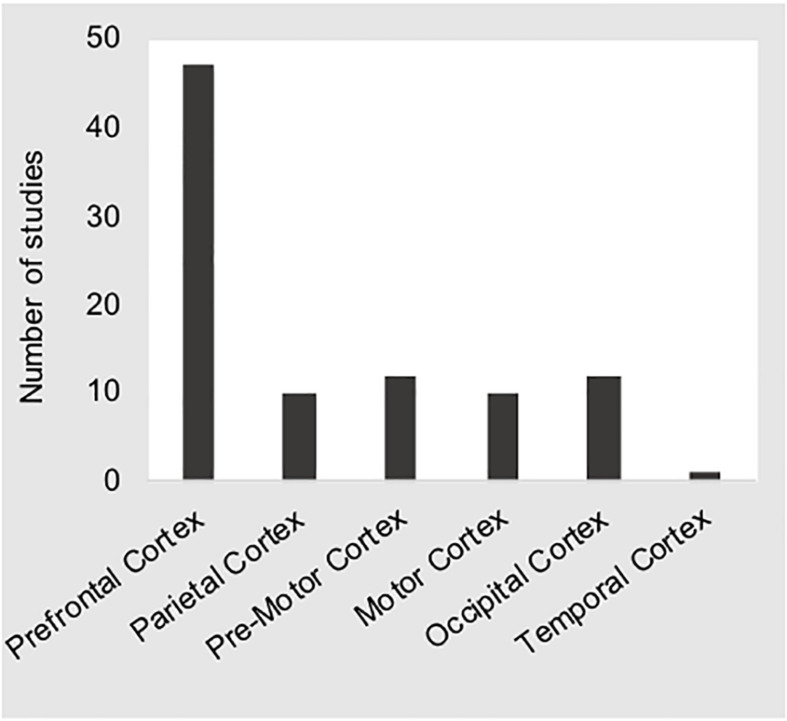
The bar chart shows the number of studies reporting coverage of the regions of interest given along the *x*-axis. These distributions show that the majority of studies sampled prefrontal cortical regions.

At the time this manuscript was prepared, we identified 28 fNIRS devices that were being marketed for human subjects research^4^. The specifications of these devices differ in many respects. For example, the number of optodes available in a given system will greatly affect the size and weight of the device. For lab-based studies where obtaining the greatest amount of cortical coverage possible is a priority, the issue of portability may not be an issue. However, for real-world studies that attempt to make use of a products’ portability, researchers must often sacrifice cortical coverage in lieu of a smaller form factor. These issues may also have an effect on aspects of fNIRS data quality due to variations in source strength (e.g., LED vs. laser light sources) or detector sensitivity (e.g., standard vs. avalanche photo diode), as well as sampling frequency (e.g., time-locked vs. simultaneous). Within the studies included here, 92% studies applied a total of 16 different commercially available devices of which only three were not portable solutions, while the remaining four studies (8%) reported use of devices that were built “in-house” (Ahn et al., 2016; Nosrati et al., 2016; Nguyen et al., 2017; Li et al., 2018). The mean number of channels available across all devices was *M* = 30.9 (*SD* = 25.8), and ranged from one (Sturman and Wiggins, 2019) and two channel solutions (Harada et al., 2007; Li et al., 2009; Liu, 2014; Nosrati et al., 2016; Horrey et al., 2017) to 98 channels (Orino et al., 2017). Two studies did not specify the number of channels used (Unni et al., 2015; Xu L. et al., 2017), and four studies reported the use of a “tandem” system to increase the number of optodes available (Shimizu et al., 2009; FakhrHosseini et al., 2015; Ihme et al., 2018; Unni et al., 2017). As depicted in Figure 4, the most common placement of optodes was over the prefrontal cortex (PFC), with 56% of all studies reporting placement solely over the PFC (Harada et al., 2007; Li et al., 2009; Tomioka et al., 2009; Tsunashima and Yanagisawa, 2009; Shimizu T. et al., 2011; Nakano et al., 2013; Inoue et al., 2014; Liu, 2014; FakhrHosseini et al., 2015; Khan and Hong, 2015; Pradhan et al., 2015; Ahn et al., 2016; Foy et al., 2016; Nosrati et al., 2016; Sibi et al., 2016, 2017; Balters et al., 2017; Horrey et al., 2017; Nguyen et al., 2017; Foy and Chapman, 2018; Huve et al., 2018, 2019; Le et al., 2018; Li et al., 2018; Khan et al., 2019; Sturman and Wiggins, 2019; Tanveer et al., 2019). Twenty-one studies (44%) report placement over the PFC as well as the motor (Yoshino et al., 2013a, b; Oka et al., 2015; Orino et al., 2015; Xu G. et al., 2017; Xu L. et al., 2017; Chuang et al., 2018), occipital (Shang et al., 2007; Shimizu et al., 2009; Unni et al., 2017; Xu G. et al., 2017; Ihme et al., 2018; Hidalgo-Munoz et al., 2019; Zhu et al., 2019), and parietal (Yoshino et al., 2013a, b; Oka et al., 2015; Orino et al., 2015; Unni et al., 2015, 2017; Bruno et al., 2018; Chuang et al., 2018; Ihme et al., 2018; Yamamoto et al., 2018, 2019; Hidalgo-Munoz et al., 2019; Zhu et al., 2019) cortices. Only one study included here did not report placement over the PFC (Lin et al., 2019), while one other study reported coverage of “almost the whole head” (Scheunemann et al., 2019).

Optode distance mediates the photon path depth that is sampled at each measurement. The recommended specification of 30–40 mm is thought to optimally sample hemodynamic activity in the cortex while maintaining an acceptable signal to noise ratio (Brigadoi and Cooper, 2015). However, because all photons pass through scalp vasculature, fNIRS measurements at the recommended optode distance are confounded by extra-cortical hemodynamics. As a solution, many fNIRS vendors now offer “short-channel” optode distances of approximately 5 mm. Since the photon path of a channel this “short” is very shallow, thus sampling only extra-cortical blood flow, much of this noise signal may be used during pre-processing or through statistical procedures to reduce unwanted artifact. Eighteen studies followed established guidelines of constant 30–40 mm distance between optodes (Harada et al., 2007; Shang et al., 2007; Shimizu S. et al., 2011; Yoshino et al., 2013a, b; Liu, 2014; Orino et al., 2015; Ahn et al., 2016; Nosrati et al., 2016; Nguyen et al., 2017; Unni et al., 2017; Bruno et al., 2018; Li et al., 2018; Hidalgo-Munoz et al., 2019; Khan et al., 2019; Lin et al., 2019; Scheunemann et al., 2019). A total of 26 articles (55%) did not, however, report optode distance, and four used varying distances, e.g., 20–30 mm (Chuang et al., 2018), 20–40 mm (Ihme et al., 2018), and 30–40 mm (Li et al., 2009). No studies included here reported the use of short-channels.

In addition to optode distance, accurate optode placement is required to target regions of interest. The use of a standardized method to place optodes is necessary to ensure that the regions of interest are appropriately covered consistently across participants. Common methods such as the International 10/20 system have been shown to provide consistent coverage despite changes in head size across participants (Okamoto et al., 2004). Outside of the needs within a study, accurate reporting of optode placement will assist in the future replication of studies. About half (52%) of the research articles reviewed here do not specify the optode placement strategy, while four studies used the 10/10 International System (Pradhan et al., 2015; Liu et al., 2017; Xu G. et al., 2017; Xu L. et al., 2017), and nine other studies the 10/20 International System (Tomioka et al., 2009; Liu, 2014; Khan and Hong, 2015; Nosrati et al., 2016; Unni et al., 2017; Bruno et al., 2018; Chuang et al., 2018; Ihme et al., 2018; Hidalgo-Munoz et al., 2019; Lin et al., 2019; Tanveer et al., 2019). Other studies just specified placement such as 4 cm from mid-line and 2 cm above supra-orbital ridge (Li et al., 2009; Shimizu T. et al., 2011; Horrey et al., 2017). Five studies used a 3D neuroscan digitizer (e.g., Polhemus^5^) to co-register the optode positions on the head (Yoshino et al., 2013a, b; Oka et al., 2015; Orino et al., 2015; Orino et al., 2017), and one study conducted a sensitivity profile by projecting the fNIRS probe onto a digital brain atlas (Foy et al., 2016). Only two studies report the use of optode placement software (Unni et al., 2017; Ihme et al., 2018).

### Data Processing and Statistical Analysis

**FIGURE 5:**
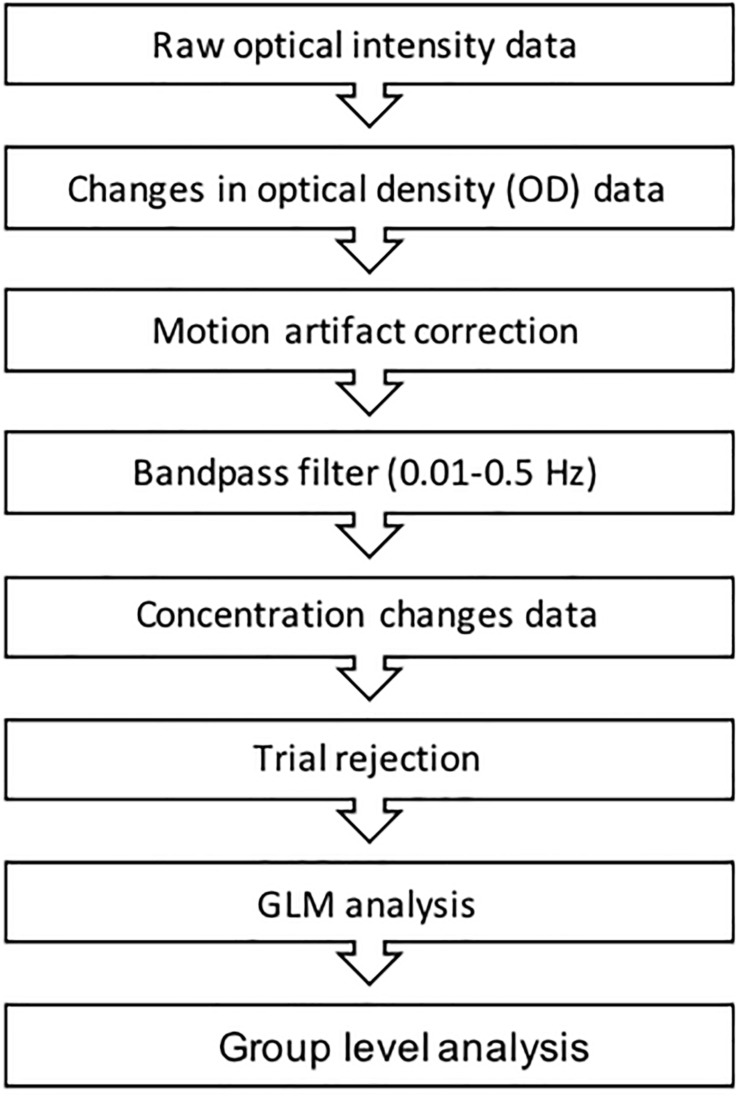
The figure above represents a common fNIRS data processing procedure.

Efforts have been made to develop and standardize fNIRS data processing procedures and tools (Brigadoi et al., 2014; Di Lorenzo et al., 2019). For example, the decision tree in Figure 5 outlines a common fNIRS data processing pipeline. We refer the reader to (Brigadoi et al., 2014) for a more detailed overview of the most common fNIRS data processing steps. While a full review of these methods is outside of the scope of this paper, the reader will see the order at which each step is generally taken, beginning with raw optical density. The attempt to standardize data processing procedures (Cutini and Brigadoi, 2014; Herold et al., 2017, 2018) has had a positive impact on the fNIRS community. Such efforts are also supported by the development and increasingly common usage of fNIRS-specific data analysis packages (e.g., HOMER2^6^, HOMER3^7^, NIRS SPM^8^, nirsLAB^9^, fNIRSOFT^10^, open-potato^11^, PHEOBE^12^, etc.).

As noted above, the method a researcher uses for motion correction is important (for review see Cooper et al., 2012; Brigadoi et al., 2014; Di Lorenzo et al., 2019). Variation in methods for motion correction is largely due to the flexibility in experimental design made possible by fNIRS, which is especially true in naturalistic research scenarios such as driving studies. The methods reported by the studies included here varied greatly including high pass filtering (Yoshino et al., 2013a, b; Unni et al., 2015; Ahn et al., 2016), low-pass filtering (Shimizu T. et al., 2011; Oka et al., 2015; Sibi et al., 2016), moving average (Tomioka et al., 2009), least square smoothing (Huve et al., 2019), Kalman filtering (Le et al., 2018), principle component analysis (Nosrati et al., 2016; Unni et al., 2017), multi-resolution analysis decomposition (Shimizu et al., 2009; Tsunashima and Yanagisawa, 2009), wavelet-based motion correction (Pradhan et al., 2015; Foy et al., 2016; Balters et al., 2017; Sibi et al., 2017; Bruno et al., 2018; Foy and Chapman, 2018; Huve et al., 2018), and Gaussian filtering (Khan and Hong, 2015; Tanveer et al., 2019). Sixteen studies did not report any filtering or artifact correction (Harada et al., 2007; Shang et al., 2007; Li et al., 2009; Nakano et al., 2013; Liu, 2014; Inoue et al., 2014; FakhrHosseini et al., 2015; Orino et al., 2015; Horrey et al., 2017; Nguyen et al., 2017; Orino et al., 2017; Yamamoto et al., 2018, 2019; Khan et al., 2019; Sturman and Wiggins, 2019; Zhu et al., 2019).

There is an ongoing discussion in the fNIRS community on which fNIRS metrics to use. Some researchers argue that it suffices to report the change in oxygenated hemoglobin (HbO) only, since HbO is assumed to be a more robust marker of changes in regional cerebral blood flow than changes in deoxygenated hemoglobin (e.g., Hoshi, 2007). Other researchers highlight the importance to report both, HbO and HbR, given that both report metrics together may provide a more complete assessment of cortical activation (e.g., Tachtsidis and Scholkmann, 2016; Herold et al., 2017). The metrics used by authors to estimate cortical activity varied between the exclusive use of HbO (Shang et al., 2007; Shimizu T. et al., 2011; Nakano et al., 2013; Liu, 2014; FakhrHosseini et al., 2015; Pradhan et al., 2015; Unni et al., 2015; Ahn et al., 2016; Balters et al., 2017; Liu et al., 2017; Sibi et al., 2017; Xu G. et al., 2017; Xu L. et al., 2017; Chuang et al., 2018; Khan et al., 2019; Lin et al., 2019), exclusive use of HbR (Foy et al., 2016; Unni et al., 2017; Scheunemann et al., 2019), or the combination of the two (Harada et al., 2007; Li et al., 2009, 2018; Shimizu et al., 2009; Tomioka et al., 2009; Tsunashima and Yanagisawa, 2009; Inoue et al., 2014; Khan and Hong, 2015; Nosrati et al., 2016; Sibi et al., 2016; Horrey et al., 2017; Nguyen et al., 2017; Bruno et al., 2018; Foy and Chapman, 2018; Ihme et al., 2018; Hidalgo-Munoz et al., 2019; Huve et al., 2019; Tanveer et al., 2019). Two studies did not report the metric used (Huve et al., 2018; Le et al., 2018). Moreover, some groups have established novel metrics such as cerebral oxygenation exchange (ΔCOE) (Li et al., 2009; Yoshino et al., 2013a, b; Oka et al., 2015; Orino et al., 2015, 2017; Yamamoto et al., 2018, 2019). For the studies reviewed here, many of the metrics used were inconsistent or not reported.

A similar variety of analysis approaches were noted for the reviewed studies. For example, the majority of studies (*N* = 29) averaged the hemodynamic activity recorded during all trials or events to calculate the mean values, standard deviations, and/or maximum values across. These values were commonly used to conduct inferential group-level statistics, including t-tests and ANOVA. The remaining approaches varied between connectivity analyses (Liu et al., 2017; Xu G. et al., 2017; Xu L. et al., 2017), GLM (Balters et al., 2017; Sibi et al., 2017; Bruno et al., 2018), linear or logistic regression on subject-level time series data (Unni et al., 2015, 2017; Ihme et al., 2018; Scheunemann et al., 2019), machine learning (Le et al., 2018; Huve et al., 2018, 2019; Khan et al., 2019; Tanveer et al., 2019; Zhu et al., 2019), factor analysis (FakhrHosseini et al., 2015), linear discriminant analysis (Khan and Hong, 2015), and frequency power analysis to conduct non-parametric test across mean change in power (Chuang et al., 2018).

## Discussion and Conclusion

**FIGURE 6:**
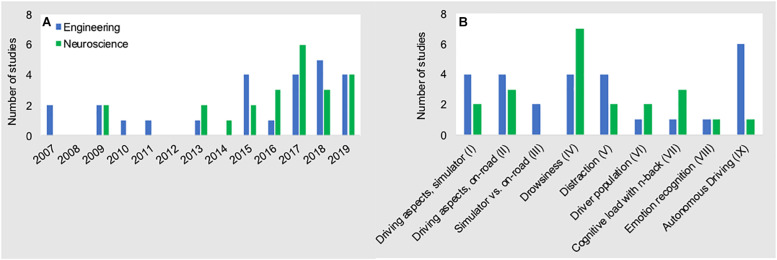
Number of fNIRS driving studies conducted from 2007 to 2019 **(A)**, and research topics **(B)** for both disciplines. Data presented here are the data that are presented in Figure 3 above, stratified across disciplines of origin.

The widespread adoption of fNIRS to study the brain’s response to driving has led to many interesting research questions and findings. This review highlighted how methodological approaches, data processing steps, and analyses often vary greatly across the studies. While such scientific diversity is well expected in this early phase of “naturalistic neuroscience,” it may also hamper the generalization of findings that allow researchers to compare and confirm results moving forward. As a consequence, systematic comparisons (i.e., meta-analysis) of the findings to establish generalizable results are difficult if not impossible. One striking outcome of our review, and one that may help explain the high amount of methodological variance across studies, is the distribution of experiments conducted across the neuroscience and engineering disciplines. As shown in Figure 6A, roughly half (*N* = 25) of the manuscripts were published in engineering journals/conference-proceedings, while the remaining manuscripts (*N* = 23) were published in neuroscience journals/conference-proceedings. Both disciplines have largely focused on the same sub-topics of driving research (see Figure 6B). This similar distribution provides a naturally occurring overlap in empirical focus across disciplines that affords a unique opportunity to compare and contrast the strengths and weaknesses of both disciplines.

**FIGURE 7:**
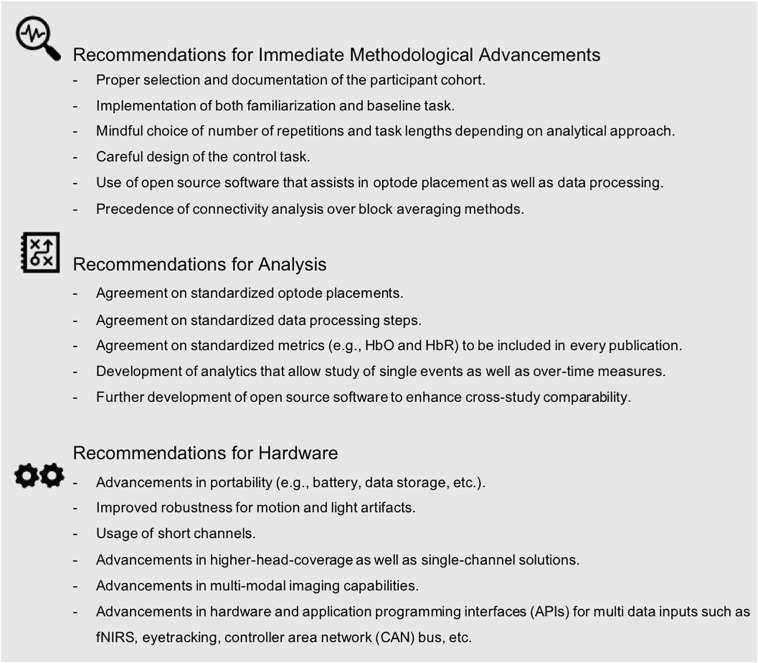
Recommendations to advance fNIRS research for autonomous driving scenarios as identified by our methodological review.

Notably, seven studies (i.e., six in engineering and one in neuroscience) applied fNIRS to assess cognitive function while driving with automated features (i.e., adaptive cruise control) (Tsunashima and Yanagisawa, 2009) or while engaging with higher automated systems (SAE Level 2–3) (Sibi et al., 2016; Balters et al., 2017; Sibi et al., 2017; Hidalgo-Munoz et al., 2019; Huve et al., 2019; Zhu et al., 2019). While these studies provide first valuable insights into brain function related to autonomous driving scenarios, the applied methodologies differed in many aspects such as analysis approach (i.e., GLM vs. block averaging vs. machine learning), data processing steps, and metrics used (i.e., HbO vs. HbO and HbR vs. tHb vs. ΔTHR), to name a few. This diversity in experimental approaches highlights the need for methodological standardization so that meta-analyses of results may be conducted in the future. Moreover, all seven autonomous driving studies were executed within a simulator environment. While exclusive use of a simulator environment might be attributed to safety-critical considerations during the experiment, the need to conduct on-road “ecologically valid” studies is obvious. Multiple factors inherent to on-road driving (e.g., motion induced artifacts due to driver movement or road imperfections, sunlight, etc.) introduce noise into fNIRS data that must be addressed to adequately analyze data from future studies. Beyond repeatability of stimuli and safety that a simulator provides, it is specifically artifacts – either induced by environment (e.g., road vibrations and sunlight) or driver (e.g., motion) that have to be mastered to advance valid driving on-the-road research. In order to move toward standardization and to “make fNIRS ready” for autonomous on-road driving, we provide recommendations below (see Figure7). We argue that an effort toward standardization and advancement within three domains (i.e., immediate methodological advancements, analysis, and hardware) may facilitate a more efficient and meaningful progression of fNIRS research toward reliable on-road measurements.

### Immediate Methodological Advancements

As described above, the field can clearly benefit from attention to participant selection and more detailed documentation of the participant cohort. Proper participant selection (e.g., sufficient sampling size) as well as proper reporting of the participant cohort will enhance research quality and generalizability, and provide the reader with the information needed for making consistent and valid inferences. Our methodological review discussed the importance of including task-familiarization procedures and the need for a physiological baseline, as well as sufficient inter-block/inter-trial intervals in future studies. We further highlighted the importance of determining task duration and task repetition, depending on the underlying analytical approach. We identified a trade-off in control task design, often driven by the desire to maintain high levels of ecological validity versus experimental control over cortical activations within a region of interest. Similarly, our review revealed considerable divergence in data processing steps across studies. Many papers did not use or did not document data filtering procedures, despite readily available processing software (e.g., Matlab-embedded HOMER 2) and step-by-step instructions (e.g., Brigadoi et al., 2014; Di Lorenzo et al., 2019). We argue that future interdisciplinary fNIRS driving research should consider using these pre-existing analysis tools. At the same time, it is vital that these open access tools are well maintained by ongoing discussions within the community to avoid detrimental biases and/or assumptions as well as stagnation in novel algorithm development. In addition, it is important that future research reports processing steps to enhance interpretation and replication. Further, our review demonstrated high variation in the hemodynamic signal used and reported. The majority of papers reported using traditional metrics of brain function (i.e., HbO and HbR), though not consistently across studies. As others have suggested (e.g., Herold et al., 2017), we propose that future studies should utilize at least the two standard fNIRS metrics (i.e., HbO and HbR). Finally, while analytical variations are expected, and indeed often required, across studies, it should be noted that differences in data processing and analysis approaches can hamper our ability to compare obtained results (Cutini and Brigadoi, 2014; Tak and Ye, 2014; Herold et al., 2017). Accurate and detailed reporting of statistical output (e.g., effect size, sample size, degrees of freedom, etc.) is essential for allowing meta-analyses of study outcomes. Of the studies included in this review, only 77% reported statistics sufficient for meta-analysis. All of the above emphasizes that a detailed reporting of methods and results is needed to enhance future research.

### Analysis

Our methodological review highlighted many different approaches with respect to the choice of data analyses, data processing, and hemodynamic proxies (e.g., oxygenated and deoxygenated) across studies. This diversity is, in part, attributed to the wide range of research questions investigated. For instance, studies focusing on single driving events (e.g., near-collision events) employed different analytical approaches than those that focused on driver fatigue over long durations. We argue that future research should utilize experimental procedures that allow for inclusion of results in future meta-analyses. Future research should also prioritize experimental approaches that are ecologically valid and analytic pipelines that can be widely adopted across researchers in all disciplines. For example, the community could agree and define standardized procedures for documentation (e.g., optode placement, data processing steps, metrics) that should be reported in publications. Journals could provide checklists, to both authors and reviewers, to ensure the reporting of necessary information. Our review further highlighted the need for the development of analytics that allow study of single events as well as over-time measures. Multi-disciplinary teams of both automotive engineers and neuroscientists could jointly tackle this challenge to acknowledge both, analytical needs for ecologically valid scenarios and neurophysiological feasibility. Further development and maintenance (along with usage) of open-source software will enhance cross-study comparability for future research. We argue that more effort is needed to develop and disseminate such analytical tools via peer-reviewed publication and open-source file sharing.

### Hardware

Further advances in fNIRS portability as well as reduction in motion artifact effects (i.e., induced by both the human and the moving environment) will undoubtedly enhance our ability to execute high-quality fNIRS autonomous driving research on the road. Reducing the effect of sunlight and/or artificial light will further increase data usability. Including short-channels in the standard set up of hardware systems will allow researchers to robustly filter and/or account statistically for noise caused by physiological signals (e.g., heart rate, mayer waves, breathing rates). Higher density head coverage with increased optodes will allow researchers to derive holistic brain models of the human cortex. Improved fit and comfort of optodes on the scalp will allow for longer study durations that may be better for long-duration driving studies. Alternatively, single-channel solutions could be applied for long duration driver monitoring scenarios once a critical brain region of interest is identified. Several studies have demonstrated multi-modal sensor approaches, such as the use of EEG-fNIRS hybrid systems, which will permit the study of brain function with a high degree of temporal and spatial resolution (Chuang et al., 2018; Lin et al., 2019). The integration of multi-modal brain imaging, along with concurrent physiological and behavioral measurements, will help to generate a more holistic and accurate model of human driving behavior. Notably, twelve of our reviewed studies already included additional sensors to detect heart rate, heart rate variability, breathing rate, as well as eye blinking and/or eye closure rate (Shimizu T. et al., 2011; Khan and Hong, 2015; Ahn et al., 2016; Horrey et al., 2017; Nguyen et al., 2017; Unni et al., 2017; Bruno et al., 2018; Chuang et al., 2018; Foy and Chapman, 2018; Lin et al., 2019; Sturman and Wiggins, 2019; Tanveer et al., 2019). These are promising examples of such a multi-modal approach.

Overall, we believe that a joint effort on the part of neuroscience and engineering disciplines will continue to advance our ability to measure and understand brain function during autonomous driving scenarios in the simulator (e.g., safe environment for critical testing) and ultimately within on-road settings. Promoting the benefits of enhanced communication and interaction between the two disciplines holds promise for motivating new and productive interdisciplinary collaborations. Potential opportunities include the convening of special interest groups at conferences, the promotion of joint-disciplinary call-for-papers and presentations, or the formation of a shared society. The interdisciplinary effort across engineering and neuroscience toward determining how the brain functions when we “operate” a motor vehicle across all SAE levels of automation, will help to design and engineer safe driving of the future.

## Author Contributions

SB: conceptualization, literature review, methodology, and writing. JB: conceptualization, methodology, and writing. JG: conceptualization and writing. AR: conceptualization, methodology, supervision, and writing. All authors contributed to the article and approved the submitted version.

## Conflict of Interest

The authors declare that the research was conducted in the absence of any commercial or financial relationships that could be construed as a potential conflict of interest.

## Footnotes

1 https://www.dmv.ca.gov/portal/vehicle-industry-services/autonomous-vehicles/autonomous-vehicle-collision-reports/

2 We include one exception: the first study, conducted in 2007, comprised a computer setup without steering wheel and pedals (Harada et al., 2007).

3 The thematic analysis was conducted individually by SB and JB. The nine topics in Figure 2B resulted from discussion and agreement based on these reviews.

4 https://alivelearn.net/?p=2752

5 https://polhemus.com/scanning-digitizing/digitizing-products/

6 https://homer-fnirs.org/

7 https://github.com/BUNPC/Homer3

8 https://www.nitrc.org/projects/nirs_spm/

9 https://nirx.net/nirslab-1

10 https://www.biopac.com/product/fnirsoft-professional-edition/?attribute_pa_size=fnir-software-professional-edition

11 https://github.com/hkwgc/open-potato/blob/master/README_EN.md

12 https://bitbucket.org/lpollonini/phoebe/wiki/Home

## References

[B1] Ahn S. Nguyen T. Jang H. Kim J. G. Jun S. C. (2016). Exploring neuro-physiological correlates of drivers’ mental fatigue caused by sleep deprivation using simultaneous eeg, ecg, and fnirs data. *Frontiers in human neuroscience* 10:219. 10.3389/fnhum.2016.00219 27242483 PMC4865510

[B2] Baker J. M. Rojas-Valverde D. Gutierrez R. Winkler M. Fuhrimann S. Eskenazi B. (2017). Portable functional neuroimaging as an environmental epidemiology tool: a how-to guide for the use of fnirs in field studies. *Environmental health perspectives* 125 094502. 10.1289/EHP2049 PMC5915206 28937962

[B3] Balters S. Sibi S. Johns M. Steinert M. Ju W. (2017). “Learning-by-doing: using near infrared spectroscopy to detect habituation and adaptation in automated driving,” in *Proceedings of the 9th International Conference on Automotive User Interfaces and Interactive Vehicular Applications (ACM)*, (New York NY: ACM), 134–143.

[B4] Begum S. (2013). “Intelligent driver monitoring systems based on physiological sensor signals: A review,” in *Proceedings of the 16th International IEEE Conference on Intelligent Transportation Systems (ITSC 2013)*, (New Jersey NJ: IEEE), 282–289.

[B5] Bosworth A. Russell M. Jacob R. J. (2019). Update of fnirs as an input to brain–computer interfaces: A review of research from the tufts human–computer interaction laboratory. *Photonics* 6 90. 10.3390/photonics6030090

[B6] Brigadoi S. Ceccherini L. Cutini S. Scarpa F. Scatturin P. Selb J. (2014). Motion artifacts in functional near-infrared spectroscopy: a comparison of motion correction techniques applied to real cognitive data. *Neuroimage* 85 181–191. 10.1016/j.neuroimage.2013.04.082 23639260 PMC3762942

[B7] Brigadoi S. Cooper R. J. (2015). How short is short? Optimum source-detector distance for short-separation channels in functional near-infrared spectroscopy. *Neurophotonics* 2:025005. 10.1117/1.NPh.2.2.025005 PMC4478880 26158009

[B8] Bruno J. L. Baker J. M. Gundran A. Harbott L. K. Stuart Z. Piccirilli A. M. (2018). Mind over motor mapping: Driver response to changing vehicle dynamics. *Human brain mapping* 39 3915–3927. 10.1002/hbm.24220 29885097 PMC6339817

[B9] Canning C. Scheutz M. (2013). Functional near-infrared spectroscopy in human-robot interaction. *Journal of Human-Robot Interaction* 2 62–84.

[B10] Chenier F. Sawan M. (2007). “A new brain imaging device based on fnirs,” in *Proceedings of the 2007 IEEE Biomedical Circuits and Systems Conference (IEEE)*, (Montreal, QC), 1–4.

[B11] Chowdhury A. Shankaran R. Kavakli M. Haque M. M. (2018). Sensor applications and physiological features in drivers’ drowsiness detection: A review. *IEEE Sensors Journal* 18 3055–3067. 10.1109/jsen.2018.2807245

[B12] Chuang C.-H. Cao Z. King J.-T. Wu B.-S. Wang Y.-K. Lin C.-T. (2018). Brain electrodynamic and hemodynamic signatures against fatigue during driving. *Frontiers in neuroscience* 12:181. 10.3389/fnins.2018.00181 PMC5881157 29636658

[B13] Cooper R. Selb J. Gagnon L. Phillip D. Schytz H. W. Iversen H. K. (2012). A systematic comparison of motion artifact correction techniques for functional near-infrared spectroscopy. *Frontiers in neuroscience* 6:147. 10.3389/fnins.2012.00147 PMC3468891 23087603

[B14] Coyle S. M. Ward T. E. Markham C. M. (2007). Brain–computer interface using a simplified functional near-infrared spectroscopy system. *Journal of neural engineering* 4 219. 10.1088/1741-2560/4/3/007 17873424

[B15] Cui X. Bray S. Bryant D. M. Glover G. H. Reiss A. L. (2011). A quantitative comparison of NIRS and fMRI across multiple cognitive tasks. *Neuroimage* 54 2808–2821. 10.1016/j.neuroimage.2010.10.069 21047559 PMC3021967

[B16] Cutini S. Brigadoi S. (2014). Unleashing the future potential of functional near-infrared spectroscopy in brain sciences. *Journal of Neuroscience Methods* 232 152–156. 10.1016/j.jneumeth.2014.05.024 24880046

[B17] Di Lorenzo R. Pirazzoli L. Blasi A. Bulgarelli C. Hakuno Y. Minagawa Y. (2019). Recommendations for motion correction of infant fnirs data applicable to data sets acquired with a variety of experimental designs and acquisition systems. *NeuroImage* 200 511–527. 10.1016/j.neuroimage.2019.06.056 31247300

[B18] FakhrHosseini M. Jeon M. Bose R. (2015). “Estimation of drivers’ emotional states based on neuroergonmic equipment: an exploratory study using fnirs,” in *Adjunct Proceedings of the 7th International Conference on Automotive User Interfaces and Interactive Vehicular Applications (ACM)*, (Nottingham), 38–43.

[B19] Fountas G. Pantangi S. S. Hulme K. F. Anastasopoulos P. C. (2019). The effects of driver fatigue, gender, and distracted driving on perceived and observed aggressive driving behavior: A correlated grouped random parameters bivariate probit approach. *Analytic methods in accident research* 22 100091. 10.1016/j.amar.2019.100091

[B20] Foy H. J. Chapman P. (2018). Mental workload is reflected in driver behaviour, physiology, eye movements and prefrontal cortex activation. *Applied ergonomics* 73 90–99. 10.1016/j.apergo.2018.06.006 30098645

[B21] Foy H. J. Runham P. Chapman P. (2016). Prefrontal cortex activation and young driver behaviour: a fnirs study. *PLoS one* 11:e0156512. 10.1371/journal.pone.0156512 27227990 PMC4881939

[B22] Goh J. Y. Goel T. Christian Gerdes J. (2020). Toward automated vehicle control beyond the stability limits: drifting along a general path. *Journal of Dynamic Systems, Measurement, and Control* 142 021004.

[B23] Harada H. Nashihara H. Morozumi K. Ota H. Hatakeyama E. (2007). A comparison of cerebral activity in the prefrontal region between young adults and the elderly while driving. *Journal of physiological anthropology* 26 409–414. 10.2114/jpa2.26.409 17641461

[B24] Herold F. Wiegel P. Scholkmann F. Mueller N. G. (2018). Applications of functional near-infrared spectroscopy (fnirs) neuroimaging in exercise–cognition science: a systematic, methodology-focused review. *Journal of clinical medicine* 7 466. 10.3390/jcm7120466 30469482 PMC6306799

[B25] Herold F. Wiegel P. Scholkmann F. Thiers A. Hamacher D. Schega L. (2017). Functional near-infrared spectroscopy in movement science: a systematic review on cortical activity in postural andwalking tasks. *Neurophotonics* 4 041403. 10.1117/1.nph.4.4.041403 PMC5538329 28924563

[B26] Hidalgo-Munoz A. R. Jallais C. Evennou M. Ndiaye D. Moreau F. Ranchet M. (2019). Hemodynamic responses to visual cues during attentive listening in autonomous versus manual simulated driving: A pilot study. *Brain and cognition* 135 103583. 10.1016/j.bandc.2019.103583 31255884

[B27] Holleman G. A. Hooge I. T. Kemner C. Hessels R. S. (2020). The reality of “real-life” neuroscience: A commentary on shamay-tsoory and mendelsohn (2019). *Perspectives on Psychological Science* 16 461–465. 10.1177/1745691620917354 32316849 PMC7961613

[B28] Horrey W. J. Lesch M. F. Garabet A. Simmons L. Maikala R. (2017). Distraction and task engagement: How interesting and boring information impact driving performance and subjective and physiological responses. *Applied ergonomics* 58 342–348. 10.1016/j.apergo.2016.07.011 27633231

[B29] Hoshi Y. (2007). Functional near-infrared spectroscopy: current status and future prospects. *Journal of biomedical optics* 12 062106. 10.1117/1.2804911 18163809

[B30] Huppert T. J. Diamond S. G. Franceschini M. A. Boas D. A. (2009). Homer: a review of time-series analysis methods for near-infrared spectroscopy of the brain. *Applied optics* 48 D280–D298. 19340120 10.1364/ao.48.00d280 PMC2761652

[B31] Huve G. Takahashi K. Hashimoto M. (2018). “fnirs-based brain–computer interface using deep neural networks for classifying the mental state of drivers,” in *Proceedings of the International Conference on ArtificialNeural Networks (Springer)*, (Cham: Springer), 353–362. 10.1007/978-3-030-01424-7_35

[B32] Huve G. Takahashi K. Hashimoto M. (2019). “Online recognition of the mental states of drivers with an fnirs-based brain-computer interface using deep neural network,” in *Proceedings of the 2019 IEEE International Conference on Mechatronics (ICM) (IEEE)*, Vol. 1 (Ilmenau), 238–242.

[B33] Ihme K. Unni A. Zhang M. Rieger J. W. Jipp M. (2018). Recognizing frustration of drivers from face video recordings and brain activation measurements with functional near-infrared spectroscopy. *Frontiers in human neuroscience* 12:327. 10.3389/fnhum.2018.00327 PMC6109683 30177876

[B34] Inoue H. Shimizu S. Takahashi N. Yoshizawa Y. Nara H. Miwakeichi F. (2014). Basic study for new assistive technology based on brain activity during car driving. *International Journal of Advanced Computer Science and Applications* 5 2014.

[B35] Khan M. J. Hong K.-S. (2015). Passive bci based on drowsiness detection: an fnirs study. *Biomedical optics express* 6 4063–4078. 10.1364/boe.6.004063 26504654 PMC4605063

[B36] Khan R. A. Naseer N. Khan M. J. (2019). “Drowsiness detection during a driving task using fnirs,” in *Neuroergonomics*, eds Ayaz H. Dehais F. (Cambridge, MA: Academic Press), 79–85. 10.1016/b978-0-12-811926-6.00013-0

[B37] Kim I. Pakdamanian E. Hiremath V. (2020). “Fundamentals and emerging trends of neuroergonomic applications to driving and navigation,” in *Neuroergonomics Cognitive Science and Technology*, ed. Nam C. (Cham: Springer), 389–406. 10.1007/978-3-030-34784-0_19

[B38] Le A. S. Aoki H. Murase F. Ishida K. (2018). A novel method for classifying driver cognitive distraction under naturalistic conditions with information from near-infrared spectroscopy. *Frontiers in human neuroscience* 12:431. 10.3389/fnhum.2018.00431 PMC6213715 30416438

[B39] Li T. Lin Y. Gao Y. Zhong F. (2018). Longtime driving induced cerebral hemodynamic elevation and behavior degradation as assessed by functional near-infrared spectroscopy and a voluntary attention test. *Journal of biophotonics* 11 e201800160. 10.1002/jbio.201800160 29978590

[B40] Li Z. Zhang M. Zhang X. Dai S. Yu X. Wang Y. (2009). Assessment of cerebral oxygenation during prolonged simulated driving using near infrared spectroscopy: its implications for fatigue development. *European journal of applied physiology* 107 281–287. 10.1007/s00421-009-1122-6 19578870

[B41] Lin C.-T. King J.-T. Chuang C.-H. Ding W. Chuang W.-Y. Liao L.-D. (2019). Exploring the brain responses to driving fatigue through simultaneous eeg and fnirs measurements. *International journal of neural systems* 30 1950018. 10.1142/s0129065719500187 31366249

[B42] Liu T. (2014). Positive correlation between drowsiness and prefrontal activation during a simulated speed-control driving task. *Neuroreport* 25 1316–1319. 10.1097/wnr.0000000000000265 25275639

[B43] Liu T. Pelowski M. Pang C. Zhou Y. Cai J. (2016). Near-infrared spectroscopy as a tool for driving research. *Ergonomics* 59 368–379. 10.1080/00140139.2015.1076057 26223971

[B44] Liu Z. Zhang M. Xu G. Huo C. Tan Q. Li Z. (2017). Effective connectivity analysis of the brain network in drivers during actual driving using near-infrared spectroscopy. *Frontiers in behavioral neuroscience* 11:211. 10.3389/fnbeh.2017.00211 PMC5671603 29163083

[B45] Lohani M. Payne B. R. Strayer D. L. (2019). A review of psychophysiological measures to assess cognitive states in real-world driving. *Frontiers in human neuroscience* 13:57. 10.3389/fnhum.2019.00057 PMC6434408 30941023

[B46] Martinho A. Herber N. Kroesen M. Chorus C. (2021). Ethical issues in focus by the autonomous vehicles industry. *Transport Reviews* 1–22. ^∗^vol, 10.1080/01441647.2020.1862355

[B47] Miller D. Sun A. Johns M. Ive H. Sirkin D. Aich S. (2015). “Distraction becomes engagement in automated driving,” in *Proceedings of the Human Factors and Ergonomics Society Annual Meeting*, Vol. 59 (Thousand Oaks, CA: SAGE Publications), 1676–1680. 10.1177/1541931215591362

[B48] Nakano Y. Kojima T. Kawanaka H. Oguri K. (2013). “Study of improving the cognitive ability of elderly drivers,” in *16th International IEEE Conference on Intelligent Transportation Systems (ITSC 2013)*, (The Hague), 547–551.

[B49] Nguyen T. Ahn S. Jang H. Jun S. C. Kim J. G. (2017). Utilization of a combined eeg/nirs system to predict driver drowsiness. *Scientific reports* 7 43933. 10.1038/srep43933 PMC5339693 28266633

[B50] Nosrati R. Vesely K. Schweizer T. A. Toronov V. (2016). Event-related changes of the prefrontal cortex oxygen delivery and metabolism during driving measured by hyperspectral fnirs. *Biomedical optics express* 7 1323–1335. 10.1364/boe.7.001323 27446658 PMC4929644

[B51] Oka N. Yoshino K. Yamamoto K. Takahashi H. Li S. Sugimachi T. (2015). Greater activity in the frontal cortex on left curves: a vector-based fnirs study of left and right curve driving. *PLoS One* 10:e0127594. 10.1371/journal.pone.0127594 25993263 PMC4438050

[B52] Okamoto M. Dan H. Sakamoto K. Takeo K. Shimizu K. Kohno S. (2004). Three-dimensional probabilistic anatomical cranio-cerebral correlation via the international 10–20 system oriented for transcranial functional brain mapping. *Neuroimage* 21 99–111. 10.1016/j.neuroimage.2003.08.026 14741647

[B53] Orino Y. Yamamoto K. Oka N. Takahashi H. Sugimachi T. Suda Y. (2017). “Relationship between brain activity and real-road driving behavior: a vector-based whole-brain functional near-infrared spectroscopy study,” in *In 9th International Driving Symposium on Human Factors in Driver Assessment, Training, and Vehicle Design*, (Manchester VT).

[B54] Orino Y. Yoshino K. Oka N. Yamamoto K. Takahashi H. Kato T. (2015). Brain activity involved in vehicle velocity changes in a sag vertical curve on an expressway: vector-based functional near-infrared spectroscopy study. *Transportation Research Record* 2518 18–26. 10.3141/2518-03 12716185

[B55] Phan D. Bab-Hadiashar A. Lai C. Y. Crawford B. Hoseinnezhad R. Jazar R. N. (2020). Intelligent energy management system for conventional autonomous vehicles. *Energy* 191 116476. 10.1016/j.energy.2019.116476

[B56] Plichta M. M. Herrmann M. J. Baehne C. G. Ehlis A. C. Richter M. M. Pauli P. (2006). Event-related functional near-infrared spectroscopy (fNIRS): are the measurements reliable? *Neuroimage* 31 116–124. 10.1016/j.neuroimage.2005.12.008 16446104

[B57] Plichta M. M. Herrmann M. J. Baehne C. G. Ehlis A. C. Richter M. M. Pauli P. (2007). Event-related functional near-infrared spectroscopy (fNIRS) based on craniocerebral correlations: reproducibility of activation? *Human Brain Mapping* 28 733–741. 10.1002/hbm.20303 17080439 PMC6871457

[B58] Pradhan A. K. Hu X.-S. F. Buckley L. Bingham C. R. (2015). “Pre-frontal cortex activity of male drivers in the presence of passengers during simulated driving: an exploratory functional near-infrared spectroscopy (fnirs) study,” in *Proceedings of the Eighth International Driving Symposium on Human Factors in Driver Assessment, Training and Vehicle Design, June 22-25, 2015*, (Utah, IA), 50–56.

[B59] Rudin-Brown C. M. Parker H. A. (2004). Behavioural adaptation to adaptive cruise control (ACC): implications for preventive strategies. *Transportation Research Part F: Traffic Psychology and Behaviour* 7 59–76. 10.1016/j.trf.2004.02.001

[B60] SAE (2018). *Taxonomy and definitions for terms related to driving automation systems for on-road motor vehicles.* Warrendale, PA: SAE International.

[B61] Scheunemann J. Unni A. Ihme K. Jipp M. Rieger J. W. (2019). Demonstrating brain-level interactions between visuospatial attentional demands and working memory load while driving using functional near-infrared spectroscopy. *Frontiers in human neuroscience* 12:542. 10.3389/fnhum.2018.00542 PMC6351455 30728773

[B62] Scholkmann F. Kleiser S. Metz A. J. Zimmermann R. Pavia J. M. Wolf U. (2014). A review on continuous wave functional near-infrared spectroscopy and imaging instrumentation and methodology. *Neuroimage* 85 6–27. 10.1016/j.neuroimage.2013.05.004 23684868

[B63] Shamay-Tsoory S. G. Mendelsohn A. (2019). Real-life neuroscience: An ecological approach to brain and behavior research. *Perspectives on Psychological Science* 14 841–859. 10.1177/1745691619856350 31408614

[B64] Shang T. Wang B. Zhang S. Wang S. (2007). “Measurement and analysis of brain activation during a driving task. In Second,” in *2ndInternational Conference on Innovative Computing, Informatio and Control (ICICIC 2007)*, (Kumamoto), 470–470.

[B65] Shimizu S. Takahashi N. Inoue H. Nara H. Miwakeichi F. Hirai N. (2011). “Basic study for a new assistive system based on brain activity associated with spatial perception task during car driving,” in *In Proceedings of the 2011 IEEE International Conference on Robotics and Biomimetics (IEEE)*, (Karon Beach), 2884–2889.

[B66] Shimizu T. Hirose S. Obara H. Yanagisawa K. Tsunashima H. Marumo Y. (2009). Measurement of frontal cortex brain activity attributable to the driving workload and increased attention. *SAE International Journal of Passenger Cars-Mechanical Systems* 2 736–744. 10.4271/2009-01-0545

[B67] Shimizu T. Nanbu T. Sunda T. (2011). An Exploratory Study of the Driver Workload Assessment by Brain Functional Imaging Using Onboard fNIRS. *Tech. rep., SAE Technical Paper* 2011 1–11. 10.1155/2009/164958 19584938 PMC2703809

[B68] Sibi S. Ayaz H. Kuhns D. P. Sirkin D. M. Ju W. (2016). “Monitoring driver cognitive load using functional near infrared spectroscopy in partially autonomous cars,” in *Proceedings of the 2016 IEEE Intelligent Vehicles Symposium (IV) (IEEE)*, (Gothenburg), 419–425.

[B69] Sibi S. Baiters S. Mok B. Steiner M. Ju W. (2017). “Assessing driver cortical activity under varying levels of automation with functional near infrared spectroscopy,” in *Proceedings of the 2017 IEEE Intelligent Vehicles Symposium (IV) (IEEE)*, (Redondo Beach, CA), 1509–1516.

[B70] Solovey E. T. Girouard A. Chauncey K. Hirshfield L. M. Sassaroli A. Zheng F. (2009). “Using fnirs brain sensing in realistic hci settings: experiments and guidelines,” in *Proceedings of the 22nd annual ACM symposium on User interface software and technology*, (Victoria, BC), 157–166.

[B71] Strangman G. Culver J. P. Thompson J. H. Boas D. A. (2002). A quantitative comparison of simultaneous BOLD fMRI and NIRS recordings during functional brain activation. *Neuroimage* 17 719–731. 10.1006/nimg.2002.1227 12377147

[B72] Sturman D. Wiggins M. W. (2019). Drivers’ cue utilization predicts cognitive resource consumption during a simulated driving scenario. *Human factors* 0018720819886765. ^∗^vol, 10.1177/0018720819886765 31721607

[B73] Tachtsidis I. Scholkmann F. (2016). False positives and false negatives in functional near-infrared spectroscopy: issues, challenges, and the way forward. *Neurophotonics* 3 031405. 10.1117/1.nph.3.3.031405 PMC4791590 27054143

[B74] Tak S. Ye J. C. (2014). Statistical analysis of fnirs data: a comprehensive review. *Neuroimage* 85 72–91. 10.1016/j.neuroimage.2013.06.016 23774396

[B75] Takahashi N. Shimizu S. Hirata Y. Nara H. Miwakeichi F. Hirai N. (2010). “Fundamental study for new assistive system based on brain activity during car driving,” in *Proceedings of the 2010 IEEE International Conference on Robotics and Biomimetics*, (New Jersey NJ: IEEE), 745–750.

[B76] Tan H. Sun J. Wenjia W. Zhu C. (2021). User Experience & Usability of Driving: A Bibliometric Analysis of 2000-2019. *International Journal of Human–Computer Interaction* 1–11. ^∗^vol,

[B77] Tanveer M. A. Khan M. J. Qureshi M. J. Naseer N. Hong K.-S. (2019). Enhanced drowsiness detection using deep learning: An fnirs study. *IEEE Access* 7 137920–137929. 10.1109/access.2019.2942838

[B78] Tao D. Zhang R. Qu X. (2017). The role of personality traits and driving experience in self-reported risky driving behaviors and accident risk among chinese drivers. *Accident Analysis & Prevention* 99 228–235. 10.1016/j.aap.2016.12.009 27984813

[B79] Tomioka H. Yamagata B. Takahashi T. Yano M. Isomura A. J. Kobayashi H. (2009). Detection of hypofrontality in drivers with alzheimer’s disease by near-infrared spectroscopy. *Neuroscience letters* 451 252–256. 10.1016/j.neulet.2008.12.059 19146927

[B80] Tsunashima H. Yanagisawa K. (2009). Measurement of brain function of car driver using functional near-infrared spectroscopy (fnirs). *Computational intelligence and neuroscience* 2009 164958. 10.1155/2009/164958 PMC2703809 19584938

[B81] Unni A. Ihme K. Jipp M. Rieger J. W. (2017). Assessing the driver’s current level of working memory load with high density functional near-infrared spectroscopy: a realistic driving simulator study. *Frontiers in human neuroscience* 11:167. 10.3389/fnhum.2017.00167 PMC5380755 28424602

[B82] Unni A. Ihme K. Surm H. Weber L. Ludtke A. Nicklas D. (2015). “Brain activity measured^..^ with fnirs for the prediction of cognitive workload,” in *Proceedings of the 20102015 6th IEEE International Conference on Cognitive Infocommunications (CogInfoCom)*, (New Jersey NJ: IEEE), 349–354.

[B83] Virtanen J. Kotilahti K. M. Ilmoniemi R. Noponen T. E. Virtanen J. (2011). Accelerometer-based method for correcting signal baseline changes caused by motion artifacts in medical near-infrared spectroscopy. *Journal of biomedical optics* 16 087005. 10.1117/1.3606576 21895332

[B84] Ware M. Feng J. Nam C. S. (2020). “Neuroergonomics behind the wheel: Neural correlates of driving,” in *Neuroergonomics*, (Cham: Springer), 353–388. 10.1007/978-3-030-34784-0_18

[B85] Winter J. D. Stanton N. A. Price J. S. Mistry H. (2016). The effects of driving with different levels of unreliable automation on self-reported workload and secondary task performance. *International journal of vehicle design* 70 297–324. 10.1504/ijvd.2016.076736

[B86] Wu C. Bayen A. M. Mehta A. (2018). “Stabilizing traffic with autonomous vehicles,” in *Proceedings of the 2018 IEEE International Conference on Robotics and Automation (ICRA)*, (New Jersey NJ: IEEE), 1–7.

[B87] Xu G. Zhang M. Wang Y. Liu Z. Huo C. Li Z. (2017). Functional connectivity analysis of distracted drivers based on the wavelet phase coherence of functional near-infrared spectroscopy signals. *PLoS one* 12:e0188329. 10.1371/journal.pone.0188329 29176895 PMC5703451

[B88] Xu L. Wang B. Xu G. Wang W. Liu Z. Li Z. (2017). Functional connectivity analysis using fnirs in healthy subjects during prolonged simulated driving. *Neuroscience letters* 640 21–28. 10.1016/j.neulet.2017.01.018 28087436

[B89] Yamamoto K. Takahashi H. Sugimachi T. Nakano K. Suda Y. (2019). The study of driver’s brain activity and behaviour on ds test using fnirs. *IFAC-PapersOnLine* 51 244–249. 10.1016/j.ifacol.2019.01.045

[B90] Yamamoto K. Takahashi H. Sugimachi T. Suda Y. (2018). “The study of driver’s reaction for traffic information on actual driving and ds using fnirs,” in *Proceedings of the 2018 IEEE International Conference on Computational Intelligence and Virtual Environments for Measurement Systems and Applications (CIVEMSA)*, (New Jersey NJ: IEEE), 1–6.

[B91] Yoshino K. Oka N. Yamamoto K. Takahashi H. Kato T. (2013a). Correlation of prefrontal cortical activation with changing vehicle speeds in actual driving: a vector-based functional near-infrared spectroscopy study. *Frontiers in Human Neuroscience* 7:895. 10.3389/fnhum.2013.00895 PMC3872330 24399953

[B92] Yoshino K. Oka N. Yamamoto K. Takahashi H. Kato T. (2013b). Functional brain imaging using near-infrared spectroscopy during actual driving on an expressway. *Frontiers in human neuroscience* 7:882. 10.3389/fnhum.2013.00882 PMC3871711 24399949

[B93] Yücel M. A. Lühmann A. V. Scholkmann F. Gervain J. Dan I. Ayaz H. (2021). Best practices for fNIRS publications. *Neurophotonics* 8: 012101. 10.1117/1.NPh.8.1.012101 PMC7793571 33442557

[B94] Zhu L. Li S. Li Y. Wang M. Zhang C. Li Y. (2019). Analysis of braking intention based on fnirs in driving simulation experiments. *IET Intelligent Transport Systems* 13 1181–1189. 10.1049/iet-its.2018.5304

[B95] Zhu Y. Rodriguez-Paras C. Rhee J. Mehta R. K. (2020). Methodological approaches and recommendations for functional near-infrared spectroscopy applications in hf/e research. *Human factors* 62 613–642. 10.1177/0018720819845275 31107601

